# 1637. Safety Evaluation of the Novel Oral Polio Vaccine Type 2 during a National Supplementary Immunisation Activity in Uganda, a Multipronged Approach: January–March 2022

**DOI:** 10.1093/ofid/ofad500.1471

**Published:** 2023-11-27

**Authors:** Fred Nsubuga, Zunera Gilani, Farrell A Tobolowsky, Ashley T Longley, Sharon A Greene, Ismail Ntale, Ndagije Hellen, Annet Kisakye, Immaculate Ampaire, Daniel Kyabayinze, Jane F Gidudu

**Affiliations:** Ministry of Health, Uganda, AFENET, Kampala, Wakiso, Uganda; Centers for Disease Control and Prevention, Center for Global Health, Global Immunization Division, Baltimore, Maryland; CDC, Atlanta, Georgia; CDC, Atlanta, Georgia; Centers for Disease Control and Prevention, Atlanta, Georgia; National Drug Authority, Kampala, Kampala, Uganda; National Drug Authority, Kampala, Kampala, Uganda; World Health Organisation, Kampala, Wakiso, Uganda; Ministry of Health, Uganda, Kampala, Wakiso, Uganda; Ministry of Health, Uganda, Kampala, Wakiso, Uganda; US Centers for Disease Control and \prevention, Atlanta, Georgia

## Abstract

**Background:**

Data from clinical trials show that novel oral poliovirus vaccine type 2 (nOPV2) is safe. However, nOPV2 is under emergency use listing, and continued vaccine safety monitoring is required to support licensure and World Health Organization (WHO) prequalification. In January 2022, Uganda introduced nOPV2 in a national supplementary immunization activity (SIA) in response to a circulating vaccine derived poliovirus outbreak, vaccinating 9.8 million children < 5 years old. We sought to identify and characterize safety events following nOPV2 vaccination

**Methods:**

We evaluated the safety of nOPV2 following the SIA in Uganda using a multi-pronged approach: 1) routine passive surveillance for adverse events following immunization (AEFI), 2) active hospital-based surveillance for adverse events of special interest (AESI) 3) active acute flaccid paralysis (AFP) surveillance, and 4) active, cohort event monitoring (CEM) among a nationally representative sample of 2000 households in 200 enumeration areas for 42 days following administration of the nOPV2 vaccine. The national AEFI committee conducted causality assessment for all reported serious AEFI and pre-specified AESI conditions using global guidelines.

**Results:**

During 14 January–11 March 2022, 1,157 adverse events were identified: 43 (3.7%) from passive surveillance, 5 (0.43%) from hospital-based surveillance, 157 (14%) from AFP surveillance, and 952 (82%) from CEM. The most commonly reported adverse events identified through CEM 0-3 days post-vaccination were fever (n=377, 40%), diarrhea (n=147, 15%), and malaise (n=107, 11%). Through all surveillance systems, 171 events underwent causality assessment. Among these 7 (4%) were classified as vaccine product-related reactions (1 acute disseminated encephalomyelitis, 1 encephalitis, 1 fever, 3 gastro-enteritis, and 1 painful lower limbs), 101 (59%) were classified as coincidental events, and 63 (37%) were downgraded.

**Conclusion:**

The majority of identified serious AEFI were classified as coincidental events. No unexpected safety signals were identified among children < 5 years old during the nOPV2 SIA in Uganda. This evaluation provides critical data to contribute to the vaccine’s safety profile and informs recommendations for use, pre-qualification, and licensure.

**Disclosures:**

**All Authors**: No reported disclosures

